# Supplementary material for: Prenyl Transferases Regulate Secretory Protein Sorting and Parasite Morphology in Toxoplasma gondii
Source: Int J Mol Sci. 2023 Apr 12;24(8):7172. doi: 10.3390/ijms24087172 (PMC10138696; doi:10.3390/ijms24087172)
Supplement: Supplementary file 1 [file ijms-24-07172-s001.zip › Supplementary Figures S1-S3.pdf]

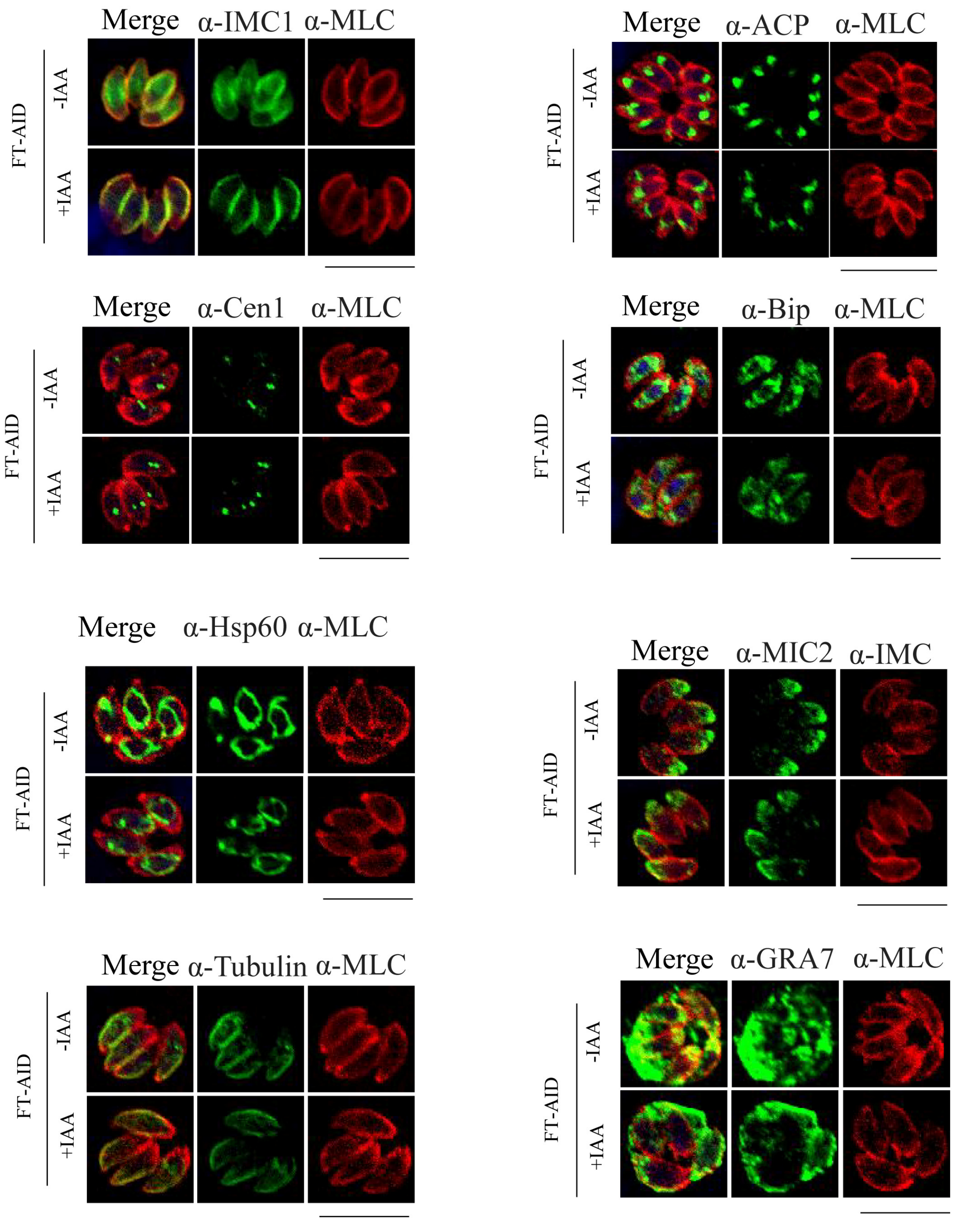

Figure S1. Observation of morphology of parasite organelles not affected by FT. IFA was executed to detect the phenotype of organelles and MLC was used to label the outer plasma of the parasite. ACP, IMC1, Cen1, Bip, Hsp60, MIC2, and Tubulin represented the organelles apicoplast, IMC, centrosome, ER, mitochondrion, microneme, and microtubes respectively. Scare bars: 10 μM.

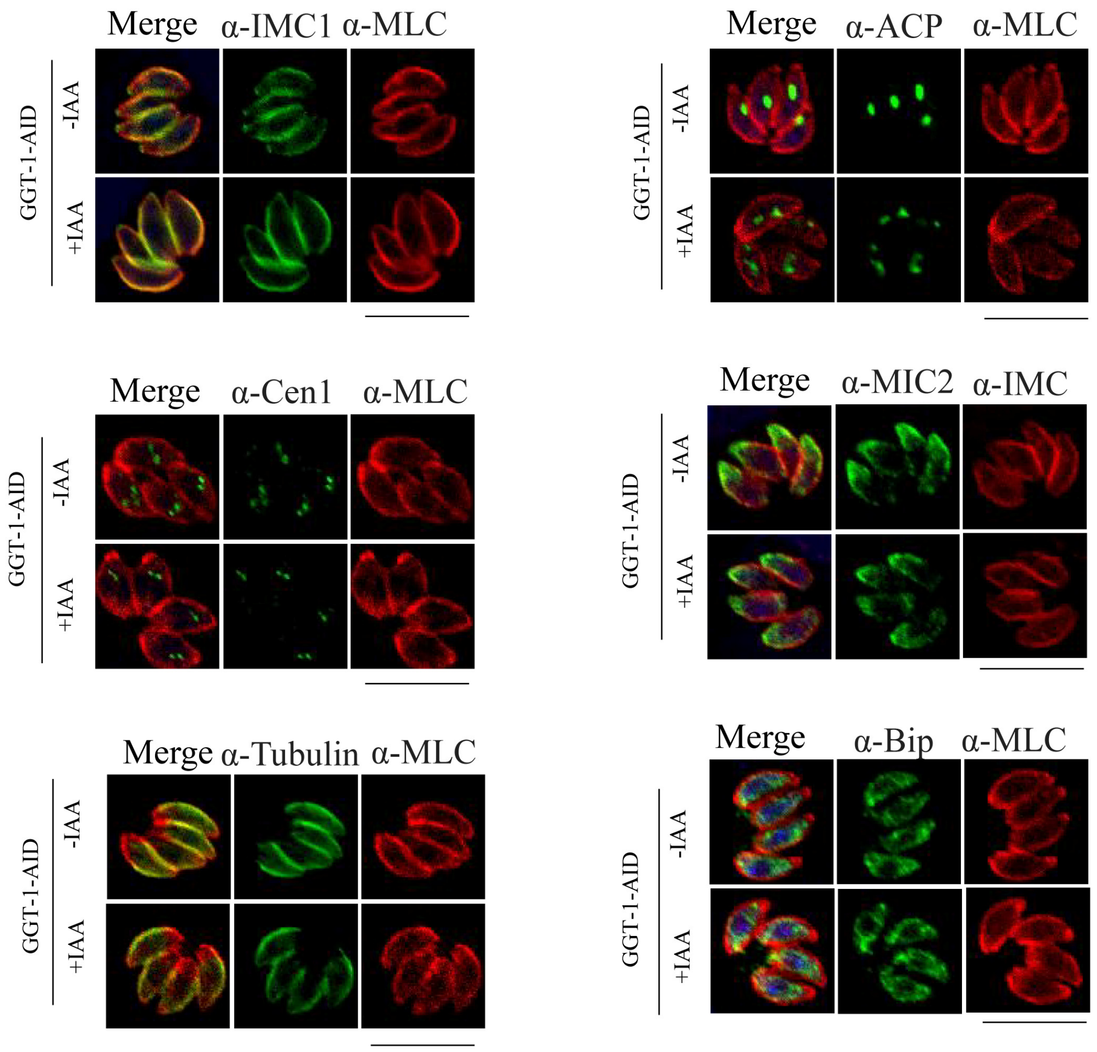

Figure S2. Observation of phenotype of parasite organelles not affected by GGT-1. The morphology of organelles was examined by IFA and MLC was utilized to label the outer plasma of the parasite. IMC1, ACP, Cen1, MIC2, Tubulin, and Bip were used as the marker of IMC, apicoplast, centrosome, microneme, microtubes, and ER respectively. Scare bars: 10 μM.

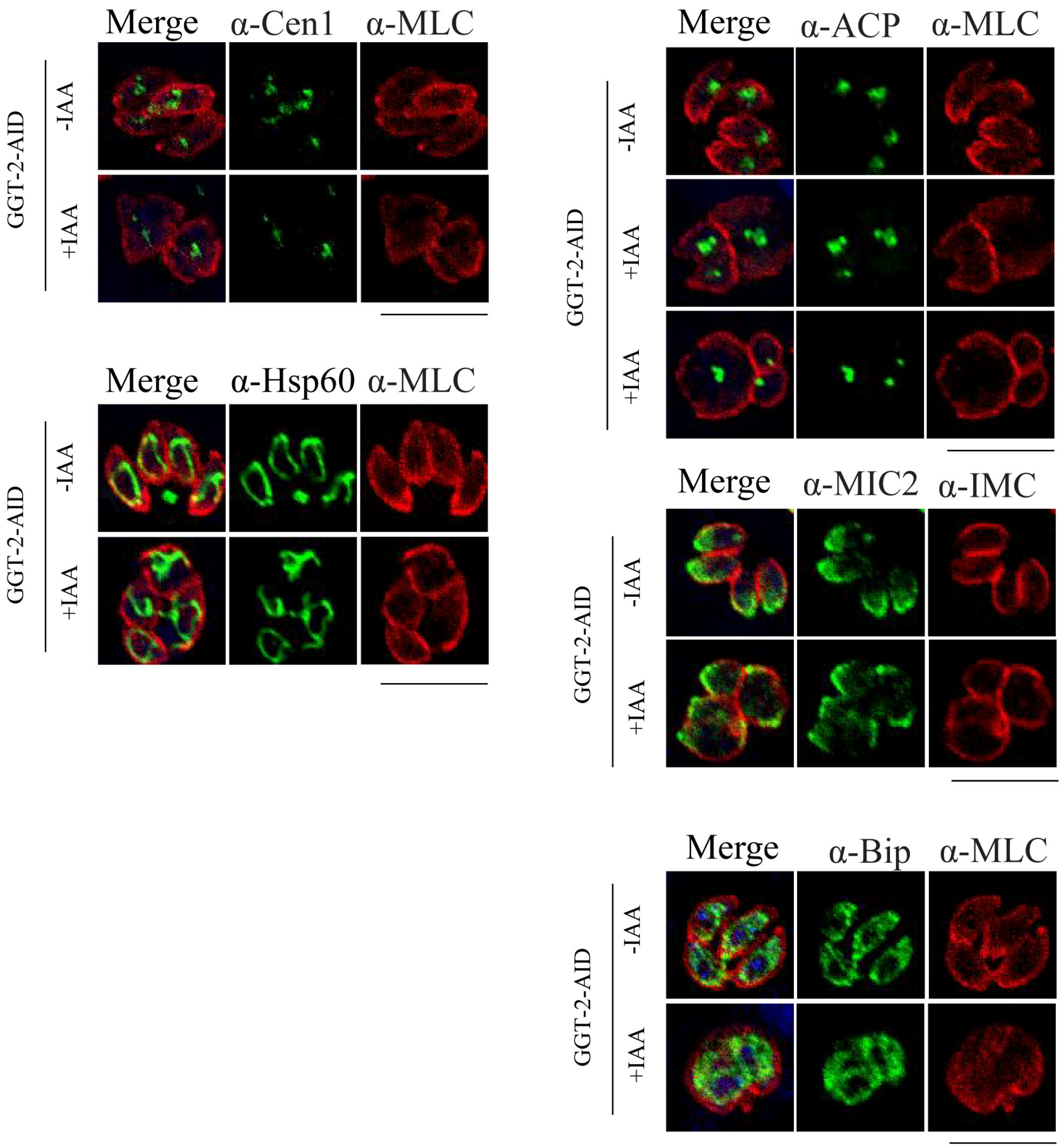

Figure S3. Observation of morphology of parasite organelles not affected by GGT-2. IFA was implemented to examine the phenotype of organelles and MLC was used to label the outer plasma of the parasite. Cen1, ACP, Hsp60, MIC2, and Bip were symbolized the centrosome, apicoplast, mitochondrion, microneme, and ER respectively. Scare bars: 10 μM.
